# Electromagnetic Shielding Performance of Carbon Black Mixed Concrete with Zn–Al Metal Thermal Spray Coating

**DOI:** 10.3390/ma13040895

**Published:** 2020-02-17

**Authors:** Han-Seung Lee, Jin-ho Park, Jitendra Kumar Singh, Hyun-Jun Choi, Soumen Mandal, Jong-Min Jang, Hyun-Min Yang

**Affiliations:** 1 Department of Architectural Engineering, Hanyang University, 1271 Sa 3-dong, Sangrok-gu, Ansan 15588, Korea; ercleehs@hanyang.ac.kr (H.-S.L.); jinho9422@naver.com (J.-h.P.); 2 Department of Living and Build Environment Research, Goyangdae-Ro, Ilsanseo-Gu, Goyang-Si, Gyeonggi-Do 10223, Korea; 3 Innovative Durable Building and Infrastructure Research Center, Department of Architectural, Engineering, Hanyang University, 1271 Sa3-dong, Sangrok-gu, Ansan 15588, Korea; guswns5026@naver.com (H.-J.C.); jangjm@hanyang.ac.kr (J.-M.J.); yhm04@hanyang.ac.kr (H.-M.Y.); 4 Intelligent Construction Automation Center, Kyungpook National University, 80, Daehak-ro, Buk-gu, Daegu 41566, Korea; sou.chm@gmail.com

**Keywords:** electromagnetic pulse, shielding effect, concrete, arc thermal spray, carbon black, aluminum, zinc

## Abstract

The electromagnetic pulse (EMP) is a destructive phenomenon which harms the building, telecommunication, and IT based infrastructure. Thus, it is required to reduce the effect of EMP using shielding materials. In the present study, we have used different thickness of concrete walls by incorporating 1 and 5 wt% of carbon black, as well as 100 µm thick Zn–Al coating using the arc thermal metal spraying method (ATMSM). The EMP was evaluated using waveguide measurement fixture for shielding performance of the concrete wall in the range of 0.85 to 1 GHz frequency. The results reveal that the maximum value, i.e., 41.60 dB is shown by the 5-300-N specimen before application of Zn–Al coating where the thickness of concrete wall was 300 mm and 5% carbon black. However, once the 100 µm thick Zn–Al coating was applied on concrete specimen, this value was increased up to 89.75 dB. The increase in shielding values around 48 dB after using the Zn–Al coating is attributed to the reflection loss of the metal thermal spray coating. Thus, the Zn–Al coating can be used for EMP application instead of metallic plate.

## 1. Introduction

Electromagnetic pulse (EMP) typically refers to high power electromagnetic (HPEM) waves ranging from 100 kHz–100 MHz which damages the building structures and military equipment [1]. Depending on its source, EMP can be classified into three categories, i.e., nuclear EMP (NEMP) caused by a nuclear explosion, non-nuclear EMP (NNEMP), or international electromagnetic interference (IEMI) in which electromagnetic waves are directly generated by electromagnetic bombs or high power electromagnetic generators, and lightning EMP (LEMP) caused by natural phenomena such as lightning and huge current flow [2]. EMPs have been investigated primarily in developed countries for applications including EMP bombs and other specific purposes. EMPs that exceed the control range have the potential to neutralize the telecommunication, IT-based infrastructures, and may cause secondary injury and causalities in a population [3]. EMP consists of a high energy signal (electronic wave) by producing 50 to 100 kV/m electric field and is able to destruct the structures around 400 km ranges [4,5]. However, in general, the radio trans-receiver can sustain up to several mV only while EMP creates very strong electromagnetic energy [5].

Therefore, the development of technology for shielding electromagnetic waves has been actively conducted with an emphasis on EMP protection facilities [6,7,8]. In particular, shielding plates are generally processed using metal such as galvanized, copper, and steel based on a welding or bolt assembly.

However, when EMP is applied in the field, deterioration of the electromagnetic shielding occurs owing to using defective metal plates, error in joint regions, and problem in connection of the welding and bolts. Moreover, inefficient utilization of space due to the distance from the wall is also a potential problem [9,10].

The electromagnetic shielding generally consists of three principles: Reflection loss, absorption loss, and multireflection loss. Reflection loss is caused by the impedance difference between the air layer and the shielding material, which is known as the main shielding principle in electromagnetic wave shielding. The second is the absorption loss, which occurs in low density, thin matching thickness, high complex permittivity values along with high conductivity, as well as magnetic materials where the electromagnetic waves pass through the shielding material and are converted into heat via ohmic loss. The third is multireflection loss, caused by electromagnetic waves propagating in a completely different direction through the materials that cannot be penetrated due to the retro-reflection or electromagnetic scattering into the shielding materials [11,12].

In general, the overall shielding effect of a metal can be expressed by the sum of the absorption loss (A), reflection loss (R), and multiple reflection loss (B), as described in Equation (1):

SE = A + R + B [dB](1)

In this equation, all terms are expressed in dB and the multireflection loss (B) can be ignored if the absorption loss (A) is greater than 9 dB. Due to the conducting nature of metals, the absorption of shielding materials is greater and the chances to bounce back the waves are less. Thus, the multireflection loss (B) between two surfaces of the metal materials is very low and can be neglected [11,12]. Absorption loss in metals can be expressed using Equation (2) as follows:

(2) $$A=0.085t\sqrt{f\mu_{r}\sigma_{r}}\;[\mathrm{dB}]$$

where *t* is the thickness (meter), *f* is the frequency (Hz), *µ_r_* is relative permeability with respect to free space, and *σ_r_* is the relative conductivity of the shielding materials with respect to copper. As shown in Equation (2), the absorption loss is proportional to the thickness of the shielding materials, and then the reflection loss in the metal can be expressed by Equation (3):

(3) $$R=168+10\mathrm{lg}\left(\frac{\sigma_{r}}{\mu_{r}f}\right)\;[\mathrm{dB}]$$

Equations (1)–(3) represent the shielding effects that are typically applied when the shielding material is metal. However, the mathematical description has not been proposed yet for metal shielding materials including concrete [13,14,15,16].

Existing studies on electromagnetic wave shielding performance (in terms of field and practical applications) can be classified into either a construction aspect (reflection loss), or a material aspect (absorption loss and multireflection loss). Lee et al. have considered and derived the construction aspect using the metallic coating by the thermal spray method that is effective for the shielding of electromagnetic waves by comparing with iron and copper plates [14]. They have found that all shielding performance of coating was satisfactorily. In terms of materials, Nam et al. [17] have investigated the electromagnetic wave shielding performance of silica fume (SF) and multiwalled carbon nanotubes (MWCNT) by incorporating them into a cement composite matrix in a frequency range of 45 MHz–18 GHz and they found that when 0.6 wt% MWCNT and 20 wt% of SF were added, the greatest shielding effect was obtained at 0.94, 1.56, and 2.46 GHz. Kim et al. [18] have mentioned that silica fume increased the mechanical and electrical properties of the MWCNT cement composite. The greatest shielding effect of SF and MWCNT is owing to the increased electrical conductivity and dispersion in cement composite matric. Khushnood et.al [19] have utilized the economical carbonized peanut shell (CPS) and carbonized hazelnut shell (CHS) in concrete to improve the shielding effectiveness in a wide frequency band (0.2–10 GHz) using a commercial dielectric probe (85070D) and network analyzer (E8361A). They have found that microlevel CPS and CHS improved the dielectric constant, dielectric loss, and shielding performance. Only 0.5% CPS increased the shielding performance by 353%, 223%, 126%, and 83% at 0.9, 1.56, 2.46, and 10 GHz frequencies, respectively. The mixing of colloidal graphite in cement paste improved the electromagnetic shielding performance [20]. The graphite colloids and 0.1 µm carbon filaments did not perform better for shielding of electromagnetic waves and it shows maximum 38 dB of transmission and 21.6 dB upon reflection [21]. The exfoliated graphite composites can be used to minimize the electromagnetic shielding [22,23]. An amount of 3.1 mm flexible graphite possesses high shielding, i.e., 130 dB is owing to the large specific surface area and large skin depth [24]. Thus, it is suggested that carbon derivatives can be used as an effective shielding material. Research on the electromagnetic shielding performance of cement mortar incorporated into steel fiber was conducted by Kim et al. [25] and they have found that when the fiber length increased or the diameter decreased, the electromagnetic shielding performance increased. However, Wen and Chung [26] have explained that when 8 µm diameter and 6 mm length stainless steel is used in cement paste, 70 dB electromagnetic shielding effectiveness was obtained in comparison to the solid piece of stainless steel, i.e., 78 dB at 1.5 GH. These previous studies are mostly based on cement pastes that do not contain aggregates. These evaluations of electromagnetic wave shielding performance were performed without considering the thickness of the specimen, which affect the electromagnetic wave shielding performance.

Therefore, it is our prudent thought to study the effect of electromagnetic shielding performance of different thickness of concrete, concrete with 1 and 5 wt% carbon black and 100 µm thick Zn–Al metallic coating deposited by arc thermal spraying process onto the concrete in the range of 0.85–1 GHz frequency using the E8356A network analyzer waveguide for the protection of national security building, military base camp, hospital, etc. from the effect of EMP. Generally, the Zn–Al coating is being used for corrosion protection of smart cities infrastructure using thermal spray techniques [27,28] but we have used it to reduce the effect of EMP on construction.

## 2. Materials and Methods

### 2.1. Experiment Overview

In this study, the experimental variables used to evaluate the electromagnetic shielding performance of a concrete wall are shown in Table 1. The experimental variables were the amount of carbon black, concrete wall thickness, and the application of Zn–Al coating.

The arc thermal metal spraying method (ATMSM) was used to address the problem of metal plate in construction for existing shielding facilities owing to the cost effectiveness. In the arc thermal metal spraying method, a metal wire rod is coming out from the nozzle of a spray gun that blows compressed air, and is dispersed and cooled on the surface of the specimen to be coated [14,28,29,30,31,32,33,34,35,36,37]. Subsequently, the specimen after spraying is allowed to quench, solidify, and laminate. This makes it possible to form a coating without causing an extreme heat effect on the surface of the specimen to be coated.

The Zn and Al (purity 99.95%) twin wires of 1.6 mm diameter were used to deposit the coating onto the concrete surface. The Zn–Al coating was applied onto the concrete surface and the thickness of deposited coating was measured with a nondestructive Elcometer456 (Shinagawa-ku, Tokyo, Japan). The coating thickness was measured at three different locations on the specimen and the average was taken. The coating thickness was found to be 100 µm (±5 µm). The coating contains 68.50% Zn and 31.50% Al after the deposition which forms the pseudo alloy coating instead of pure metal.

The thickness of the concrete wall was varied from 100–300 mm with carbon black (0, 1, and 5 wt%) as the conductive material and filled the porosity of the concrete. The Zn–Al coating onto the concrete surface was deposited to evaluate the electromagnetic shielding performance. We have performed the experiment in triplicate for each set. The compressive strength of concrete specimens was performed according to the KS F 2405 standard after 28 days of curing [38]. In the case of the slump, the target value was set at 150 ± 25 mm.

### 2.2. Materials and Concrete Mixing

According to the KS L 5201 standard, we have used 3.15 g/cm^3^ density of ordinary Portland cement [39]. The average diameter of carbon particle was 23 (± 2) nm with a 120 m^2^/g specific surface area. The dibutyl phthalate (DBP) oil was used for proper dispersion of carbon particles. The chemical composition of the binder for the present study is shown in Table 2. The maximum dimension of the coarse aggregate and fine aggregates was 25 and 5 mm, respectively. To ensure the workability, 1.5% polycarboxylic superplasticizer (SP) was used.

Table 3 shows the concrete formulations used in this study. The mixing table was classified depending on the mixing ratio of the carbon black. The carbon black (purity 99.50%) was purchased from RongXin carbon products, China and it was used in 1 and 5 wt% during the mixing of concrete. The amount (wt%) of carbon black was mixed in concrete with respect to cement. As the amount of carbon black was increased, i.e., 5%, there was a difficulty in mixing of concrete owing to greater water absorption by carbon black, thus, we have added 5.4 kg dispersant. The 0.495 water/binder ratio was taken to fabricate the concrete specimens.

### 2.3. Specimen Fabrication and Curing Methods

A rectangular specimen of 100 × 200 mm was prepared in accordance with KS F 2403 [40] to measure the compressive strength of the concrete. Curing was performed using a thermo-hygrostat chamber at 20 (± 2) °C and 60 (± 2) % relative humidity for 28 days. The thickness of the specimen was set to 100, 200, and 300 mm to evaluate the applicability of the concrete for the shielding of electromagnetic waves depending on the wall thickness. After fabrication of concrete specimens, 100 (± 5) µm of Zn–Al coating was applied using the arc thermal spray coating process.

**Figure 1:**
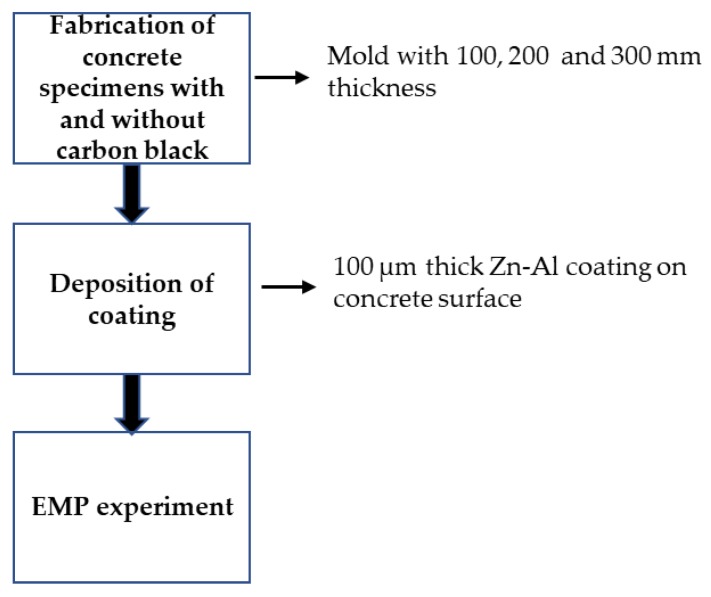
Schematic of experimental procedure.

The details of the experiment design and methodology is shown in Figure 1. The different thickness of concrete mold was fabricated using the mixture composition as shown in Table 3 with and without carbon black followed by curing for 28 days. Once the curing was completed, 100 µm thick Zn–Al coating was deposited. The electromagnetic pulse (EMP) experiment was performed for all specimens after fabrication of concrete specimens.

### 2.4. Characterization of Zn–Al Coating

The surface morphology and cross-sectional image of the coatings was carried out by scanning electron microscopy (SEM, Philips XL 30, North Billerica, MA, USA) operated at 15 kV. The cross-sectional image of the sample was taken by mounting the sample in a holder, polishing and fixing it.

The phase identification of the coating was performed by X-ray diffraction (XRD, Rigaku, Germany) using Cu Kα radiation (λ = 1.54059 Å) generated at 40 kV and 100 mA from 2θ = 10–90° at a 4°/min scan rate.

### 2.5. Electromagnetic Shielding Performance Evaluation Using Waveguide

The electromagnetic shielding performance evaluation methods for EMP protection facilities are typically known as MIL-STD-188-125-1 and IEEE-STD-299 [9,10]. However, this measurement is not easy because the type of antenna, amplifier, and the distance between the antenna and the test body which needs to be changed depends on the frequency range. Although ASTM D 4935-10 [41] is an evaluation method which uses a single coaxial cable. In this standard, the size of the specimen is fixed to a circle with 33 mm diameter which is the limit to evaluate the applicability of the concrete wall.

**Figure 2:**
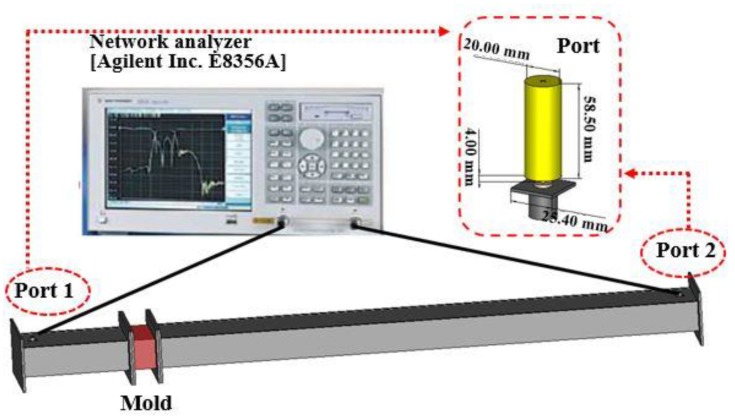
Experimental setup for electromagnetic shielding performance evaluation [42].

In this study, we have selected the E8356A network analyzer (Agilent) and a customized WR-975 waveguide having 247.65 × 123.83 mm dimension to evaluate the EMP shielding performance of the concrete wall. This wave guide is easy to set the antenna, amplifier, and the distance between the antenna and the test body. The experimental outline is shown in Figure 2 [42]. This figure shows the equipment setup for the measurement of the electromagnetic shielding.

The source of EMP was alternative current (AC) with cutoff frequency in the range of 0.85 to 1.0 GHz [9,10]. The specimen was positioned between Port 1 and Port 2 (Figure 2), then the waveguide and specimen were combined with bolts for measurement. It is possible to change Port 1 depending on the thickness of the mold.

The electromagnetic shielding performance is the signal transmitted from Port 1 and received at Port 2, which represents the intensity of the signal transmitted through the specimen, located between the waveguides. This can be expressed in Equation (4), where P1 is the power with the material and P2 is the power without the material [43,44,45]. It is measured on one length of the waveguide.

(4) $$\mathrm{SE}=10\mathrm{lg}\frac{P_{2}}{P_{1}}\;[\mathrm{dB}]$$

The minimum requirement for electromagnetic shielding in EMP protection facilities should be at least 80 dB in the target frequency band and the transmission coefficient should be measured at 80 dB or more in the network analyzer [9].

## 3. Results and Discussion

### 3.1. Concrete Material Characteristics

**Figure 3:**
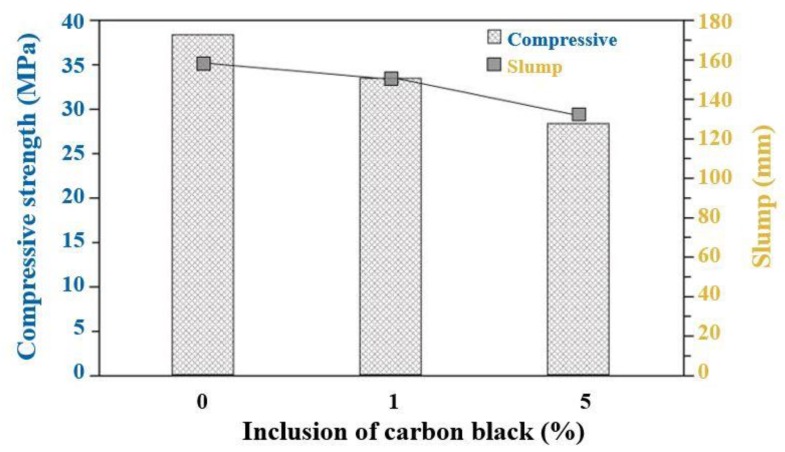
Compressive strength and slump of concrete.

The compressive strength of the concrete specimen with inclusion of carbon black was measured three times after 28-days of curing. The measured values of the slump and compressive strength are shown in Figure 3. The slump satisfies the target value of 150 ± 25 mm, and compressive strength is found to be 38.6 (± 1.93), 33.8 (± 1.35), and 28.9 (± 1.44) MPa for 0, 1, and 5 wt% inclusion of carbon black specimen, respectively.

The compressive strength tended to decrease with an increasing amount of carbon black, which is related to the properties of this material. The carbon black in the cement affects the hydration reaction by blocking the contact between water and cement. Particularly for carbon black with a large surface area, they absorb a considerable amount of water during the mixing process. As a result of these factors, the compressive strength and slump of concrete containing carbon black is reduced as the incorporation rate of carbon black increased [46].

### 3.2. SEM and XRD of Zn–Al Coating

**Figure 4:**
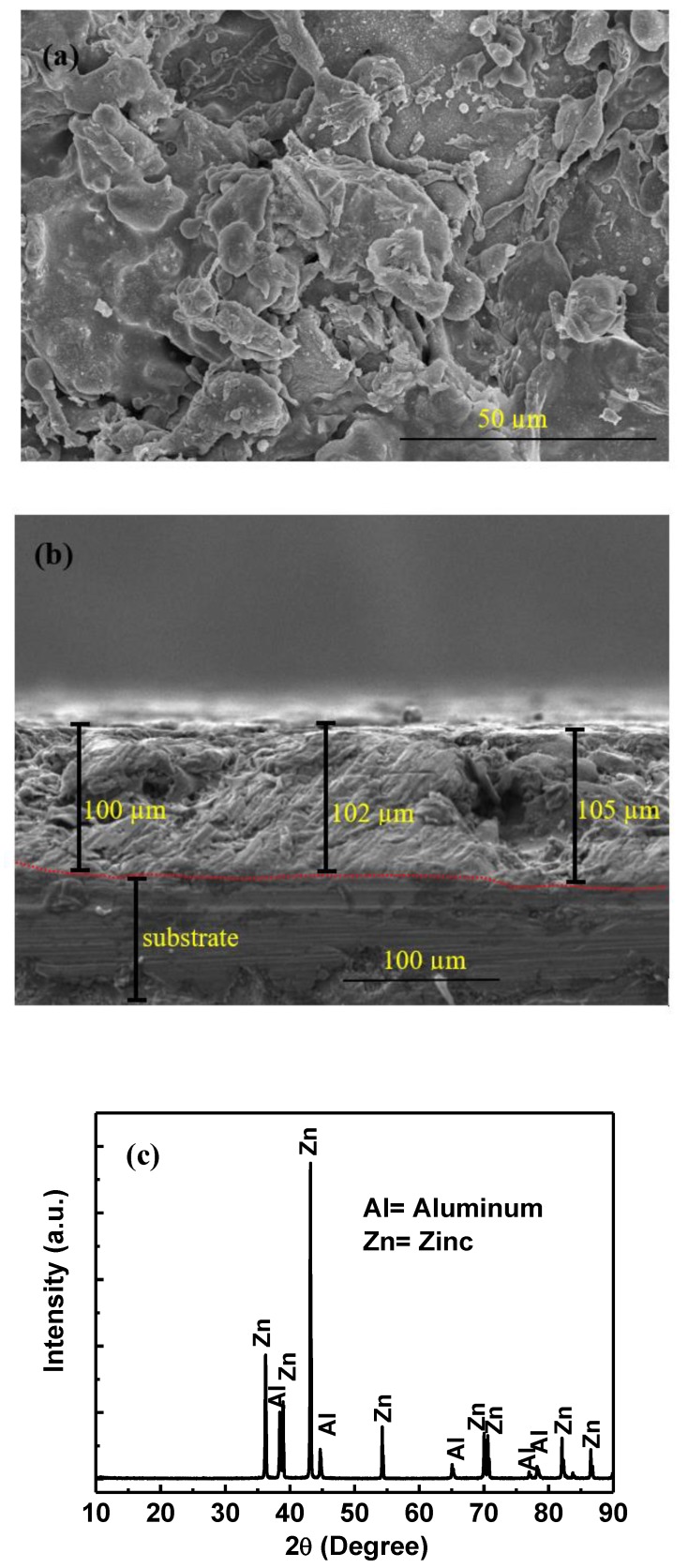
(**a**) SEM of top surface, (**b**) SEM of cross section, and (**c**) XRD of Zn–Al coating.

The SEM result of the Zn–Al coating is shown in Figure 4a. The top surface of coating exhibits splat, pores, and cracks which is an inherent property of the arc thermal spray coating process [28]. However, this coating is compact and adherent with the surface. The cross-sectional image (Figure 4b) shows around 100 (± 2) µm thickness of Zn–Al coating which verifies the result of nondestructive Elcometer456 technique. The cross-sectional image (Figure 4b) also shows cracks and defect in coating.

The presence of phases was determined by XRD and results are shown in Figure 4c. The XRD results show that the Zn–Al coating mainly contain Zn and Al (Figure 4c) phases which is a parent metal used for the deposition of the coating [28]. There is no other phase found rather than Zn and Al as revealed by XRD.

### 3.3. Evaluation of Electromagnetic Wave Shielding Performance of the Concrete

**Figure 5:**
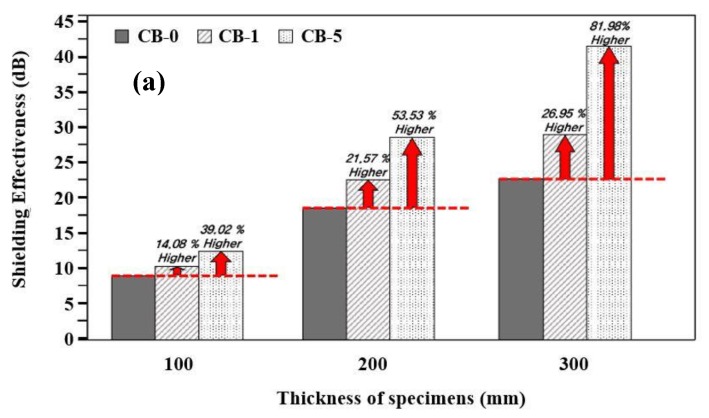

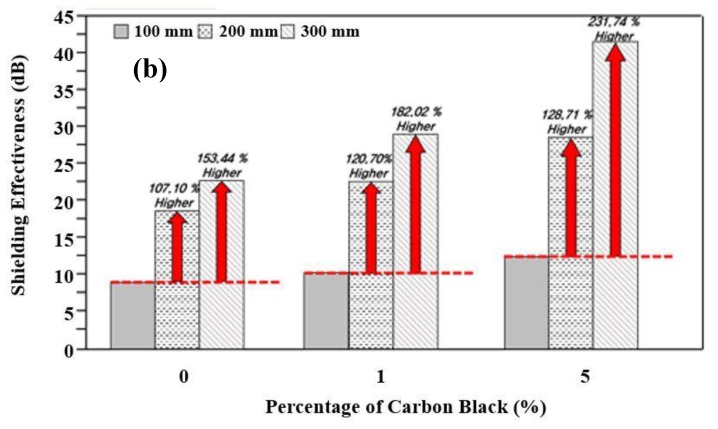
Effect of (**a**) carbon content and (**b**) concrete thickness on EMP performance of specimens before application of ATMSM coating.

The electromagnetic shielding performance of the concrete specimens with different amounts of carbon black at a particular thickness is shown in Figure 5a, while Figure 5b show the results of a fixed amount of carbon black at different thickness before application of the Zn–Al metal thermal spray coating.

From Figure 5a, it is observed that as the mixing ratio of carbon black for each thickness, i.e., 100, 200, and 300 mm of the concrete is increased, the shielding performance of the specimens increased. This result indicates that as the incorporation rate of the carbon black increases, the absorption loss also increases, resulting in an improvement of the shielding performance. The increased value of electromagnetic shielding is the highest for 300 mm with addition of 5 wt% carbon black while the lowest is found for 100 mm concrete without addition of carbon black, i.e., 9.02 dB. The electrical conductivity of the concrete incorporated with carbon black is largely affected by the formation of the conductive path inside the concrete [47]. As the thickness of the concrete increases with carbon black, the shielding performance increases. It is owing to the increase in the total amount of carbon black for the same thickness. It indicates that this effect is caused by an increase in the ratio of absorption loss due to carbon black.

When the thickness of the concrete specimens is increased by incorporating carbon black from 0, 1, and 5 wt%, the electromagnetic shielding performance increased by 107.10% to 153.44% without carbon black, 120.70% to 182.02% with 1% carbon black, and 128.71% to 231.74% with 5% carbon black for 200 to 300 mm, respectively compared to 100 mm thickness of concrete (Figure 5b). For the same carbon black incorporation rate, it is considered that the electromagnetic wave shielding performance increases as the concrete thickness increases according to the principle of absorption loss, as shown in Equation (2). It is also confirmed that the shielding performance increases as the mixing ratio of carbon black increases, as well as the increase in the thickness of the concrete.

By comparing Figure 5a,b, it is evident that the shielding performance of concrete in regards to thickness is higher compared to the incorporation of carbon black. As such, a thicker wall would give improved electromagnetic wave shielding performance but there is some limitation owing to economic viability and workability where concrete thickness cannot be increased beyond the standard. In addition, when it is associated with the workability of concrete mixed with higher amount of carbon black, it shows a negative effect for practical applications.

Therefore, to achieve acceptable performance of the concrete while considering factors such as cost and workability in addition to the thickness of the concrete, additional elements such as high-strength concrete with a dense structure or the incorporation of conductive materials should be considered. When the conductive material is mixed, it is considered to be effective in improving the electromagnetic shielding performance.

In this study, the 5-300-N specimen exhibits the highest shielding performance, i.e., 41.60 dB. However, the possibility of using carbon black mixed concrete in EMP protection facilities is low. Thus, referring to CISPR 16-2-3 and IEC 61000-4-20, in terms of the standards for electromagnetic compatibility of household appliances and lighting equipment, an average of 40 dB is defined as the allowable standard. Therefore, 5-300-N is suitable for commercial electromagnetic shielding facilities instead of EMP protection facilities [48,49].

### 3.4. Shielding Effectiveness of Specimen without Zn–Al Coating at Different Studied Frequency

**Figure 6:**
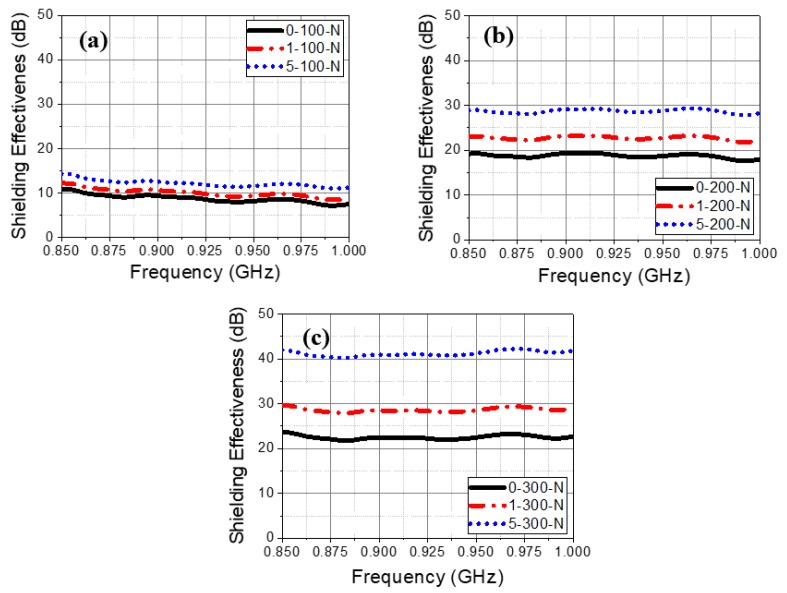
Shielding effectiveness vs. studied frequencies plots without using Zn–Al coating for (**a**) 100 (**b**) 200, and (**c**) 300 mm concrete thickness with 1 and 5 wt% carbon black.

The shielding effectiveness of the concrete specimen is increased with thickness and addition of carbon black from 0.85 to 1 GHz studied frequency range (Figure 6). However, it can be seen from Figure 6a that the shielding effectiveness is found to be lowest for 100 mm concrete thickness compared to 200 (Figure 6b) and 300 mm (Figure 6c) with and without addition of carbon black. There is little fluctuation in the shielding effectiveness observed from 0.85 to 1 GHz frequency while carbon was not added. It is owing to the semi-conducting properties of concrete where absorption loss is caused. However, once the amount of carbon black is increased from 1 to 5 wt%, the fluctuation in shielding effectiveness is minimized owing to low density and high complex permittivity of carbon which creates the conductive path for penetration of incident waves. In 100 mm concrete thickness the shielding effectiveness varies from around 11 dB at 0.85 GHz to 7 dB at 1 GHz while 200 and 300 mm concrete thickness is found to be around 20 to 17.5 dB and 24 to 21 dB at different frequency, respectively. Thus, it can infer that fluctuation is observed but once carbon was added, a straight line in shielding value is found from 0.85–1 GHz.

### 3.5. Evaluation of Electromagnetic Shielding Performance of Zn–Al Metal Thermal Spray Coating

**Figure 7:**
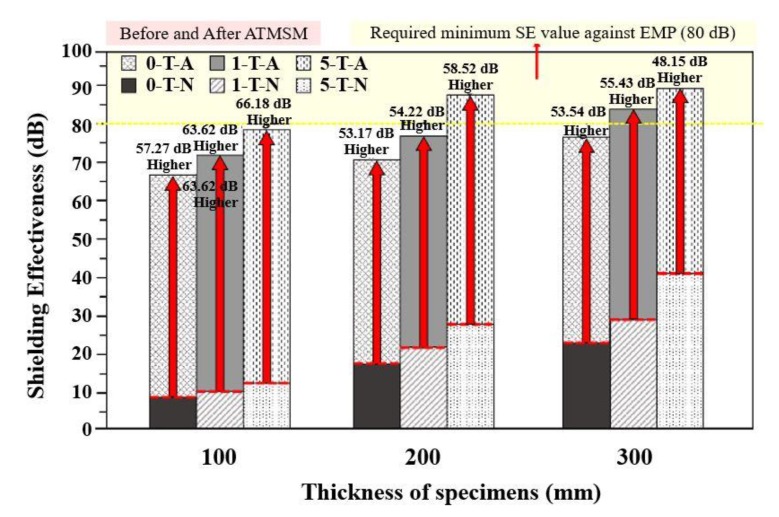
Electromagnetic shielding performance after application of the Zn–Al metal thermal spray coating.

Figure 7 shows the result of electromagnetic shielding performance after application of Zn–Al metal thermal spray coating onto the concrete surface. In legend of this figure, T, A, and N represent the thickness of the concrete specimens, presence and absence of Zn–Al coating, respectively. The specimens which satisfy the target value of 80 dB exhibited a shielding performance of 87.2, 84.45, and 89.75 dB for 5-200-A (5 wt% carbon black + 200 mm thick concrete + 100 µm Zn–Al coating), 1-300-A (1 wt% carbon black + 300 mm thick concrete + 100 µm Zn–Al coating), and 5-300-A (5 wt% carbon black + 300 mm thick concrete + 100 µm Zn–Al coating), respectively. The lowest shielding performance is found at 66.29 dB for the 0-100-A (0 wt% carbon black + 100 mm thick concrete + 100 µm Zn-Al coating) specimen. Considering the workability of the concrete incorporating carbon black, it is appropriate to apply the electromagnetic shielding facility of the 1-300-A specimen for the shielding of EMP.

The electromagnetic shielding performance of 5-300-N (5 wt% carbon black + 300 mm thick concrete + without Zn–Al coating) specimen with the highest shielding value before the metal thermal spray application was 41.60 dB, which is around 48 dB lower than after the application of Zn–Al metal coating, i.e., 89.75 dB. The Zn–Al coating was applied on the top surface of concrete where it allows to absorb the waves but due to incident of waves through the atmosphere, there would be an impedance between coating and air layer which causes the reflection loss. This result indicates that the reflection loss is mainly used for shielding of electromagnetic waves. Thus, it is suggested that the combination of reflection and absorption loss should be appropriately used to satisfy the target shielding performance of 80 dB.

As such, when a Zn–Al metal thermal spray coating is applied on 5-200-A, 1-300-A, and 5-300-A concrete specimen, a shielding value more than 80 dB was obtained. Therefore, to achieve an electromagnetic shielding performance of 80 dB, as required for an EMP protection facility, the use of a concrete containing a conductive material is necessary.

**Figure 8:**
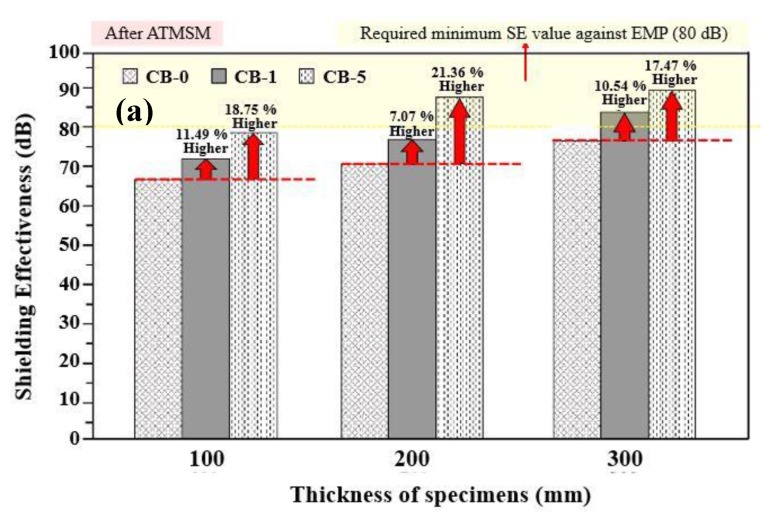

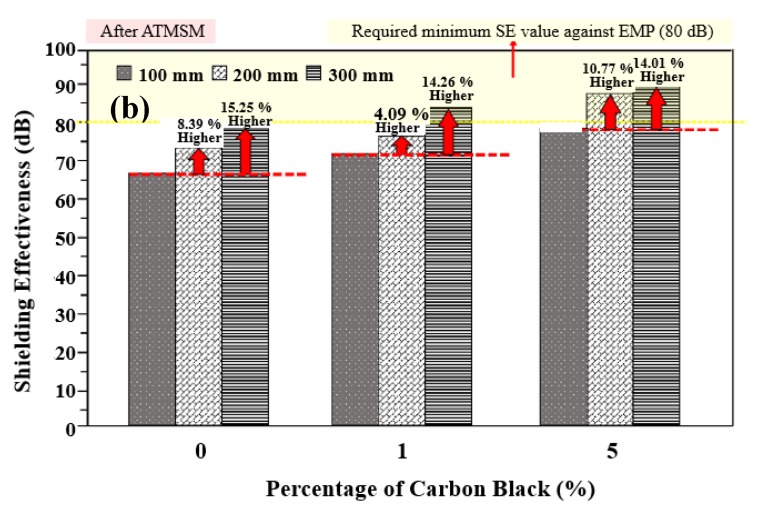
EMP performance of specimens after application of the Al–Zn coating (**a**) at a particular thickness with 0, 1, and 5 wt% carbon black and (**b**) with a fixed amount of carbon black at different thickness of concrete.

As shown in Figure 8a, after application of the Zn–Al metal thermal spray coating onto the concrete surface, the shielding performance also increased with the incorporation of carbon black for each thickness. Further, in Figure 8b, it is confirmed that the electromagnetic shielding performance increases as the thickness of concrete increases at a fixed amount of carbon black. It is considered that the shielding performance is being increased by the principle of absorption loss according to Equation (2) which is attributed owing to the mixing of carbon black and the thickness of the concrete.

In Figure 5, before the application of Zn–Al metal thermal spray, it is revealed that the improved electromagnetic shielding performance is caused owing to the thickness of concrete and content of carbon black. However, in Figure 8a,b, the rate of increase in shielding performance is relatively constant. It is attributed owing to the incident of electromagnetic waves which are significantly attenuated due to reflection loss of the metal spray coating and secondary loss due to the thickness of the concrete and carbon black. Thus, the effect of the thickness and the carbon black is reduced after the application of the Zn–Al coating on the top surface of the concrete.

### 3.6. Shielding Effectiveness of Specimen with Zn–Al Coating at Different Studied Frequency

**Figure 9:**
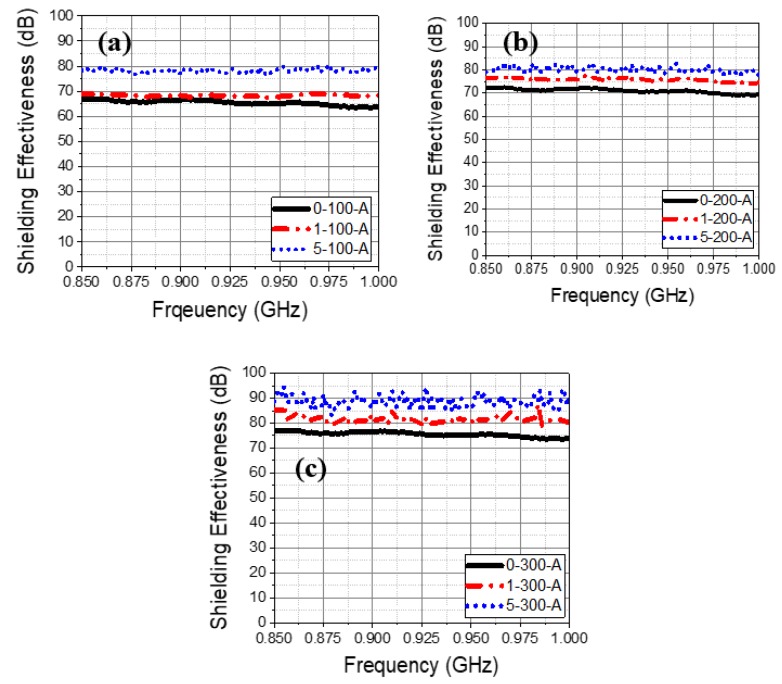
Shielding effectiveness vs. studied frequencies plots using the Zn–Al coating for (**a**) 100, (**b**) 200, and (**c**) 300 mm concrete thickness with 1 and 5 wt% carbon black.

The shielding effectiveness of the concrete specimen at different studied frequency after application of the Zn–Al coating is shown in Figure 9. It can be seen that once the coating was applied, the shielding effectiveness is increased with concrete thickness and carbon black addition owing to high complex permittivity and attenuation of electromagnetic waves within the specimen. The required shielding protection value, i.e., 80 dB is obtained by 5-200-A, 1-300-A, and 5-300-A specimens. As the thickness and carbon content is increased, the shielding effectiveness is increased. Thus, it is suggested that Zn–Al coating can be used as EMP protection facility by adding carbon black in the concrete.

## 4. Conclusions

From the above results and discussion, it is found that as the carbon content and thickness of concrete wall increased, the shielding performance increased without application of Zn–Al metal coating. Thus, the 5-300-N specimen, i.e., 5 wt% carbon black + 300 mm concrete wall thickness without Zn–Al coating exhibits 41.60 dB shielding value. Moreover, it can be only considered for application to commercial electromagnetic wave shielding facilities. However, once the Zn–Al metal thermal spray coating was applied onto the concrete surface, the shielding performance increased by 89.75 dB for 5-300-A, which is attributed owing to the reflection loss of the Zn–Al metal thermal spray coating. Thus, this process can be recommended for application of EMP. From the results, it is expected that a better shielding performance will be achieved if a hybrid system such as conductive concrete including carbon nanotubes, graphene, and steel fibers can be used by thermal spray technology.

## Figures and Tables

**Table 1:** Experimental variables.

Specimen Name	Carbon Black Content (%)	Concrete Thickness (mm)	Application of 100 µm Zn–Al Coating
0-100-N	0	100	No
0-100-A	0	100	Yes
0-200-N	0	200	No
0-200-A	0	200	Yes
0-300-N	0	300	No
0-300-A	0	300	Yes
1-100-N	1	100	No
1-100-A	1	100	Yes
1-200-N	1	200	No
1-200-A	1	200	Yes
1-300-N	1	300	No
1-300-A	1	300	Yes
5-100-N	5	100	No
5-100-A	5	100	Yes
5-200-N	5	200	No
5-200-A	5	200	Yes
5-300-N	5	300	No
5-300-A	5	300	Yes

**Table 2:** Chemical composition of binder.

% Age of Chemicals	Cement	Carbon Black
C	-	99.5
SiO_2_	19.47	0.06
Al_2_O_3_	5.24	0.03
MgO	3.72	-
SO_3_	2.49	0.31
CaO	61.8	0.04
Fe_2_O_3_	2.69	0.04
Na_2_O	0.18	-
K_2_O	0.87	-
ZnO	-	0.02
etc.	0.94	-
Loss of Ignition	2.6	-

**Table 3:** Concrete mix proportions.

Wt% of carbon black		**0%**	**1%**	**5%**
water/binder ratio		0.495	0.495	0.495
**Unit weight (kg/m^3^)**	Water	173	173	173
	Cement	350	350	350
	Carbon black	0	4	18
	Sand	873	869	856
	Gravel	929	925	912
Dispersant (kg)		-	1.2	5.4
Admixture (kg)		5.25	5.31	5.52
